# Management of Chronic Myeloid Leukemia and Pregnancy: A Bibliometric Analysis (2000-2020)

**DOI:** 10.3389/fonc.2022.826703

**Published:** 2022-03-07

**Authors:** Yue Wang, Liqing Jiang, Baoxuan Li, Yan Zhao

**Affiliations:** ^1^ Department of Obstetrics and Gynaecology, Shengjing Hospital of China Medical University, Shenyang, China; ^2^ Department of Medical Oncology, The First Affiliated Hospital of China Medical University, Shenyang, China

**Keywords:** chronic myeloid leukemia, bibliometric analysis, pregnancy, keyword analysis, fertility preservation

## Abstract

**Background:**

Given the increasing number and survival rates of reproductive-age patients with chronic myeloid leukemia (CML), several studies aimed to elucidate optimum disease management in pregnancy. This study aimed to use bibliometric analysis to assess focus and reported insights, as well as future trends, in CML and pregnancy research.

**Methods:**

We extracted all studies related to CML and pregnancy from the Web of Science database from 2001 to 2020. VOS Viewer, CiteSpace, Python, and R-bibliometrix were used for bibliometric analysis, revealing the leading research countries, institutions, and authors, as well as distribution of keywords (frequency greater than five).

**Results:**

A total of 196 records, published in 137 journals by 1,105 authors from 421 research institutes in 50 countries, were identified for analysis. The United States was the leader in the number of publications. Imperial College London and National Research Center for Hematology were the most influential institutions. In addition, Apperley J, Cortes J, Abruzzese E and Kantarjian H were the leading authors in the field. Keyword analysis identified four research hotspot clusters.

**Conclusions:**

This study systematically analyzed the progress in CML and pregnancy research in the last 20 years. The present findings suggest that the management of planned and unplanned pregnancies in patients with CML will remain a research focus, as further evidence is required for the development of treatment guidelines.

## Introduction

Chronic myeloid leukemia (CML) is a malignant clonal disease originating from hematopoietic stem cells characterized by the production of large numbers of myeloid cells at various stages of maturation in peripheral blood. The disease is characterized by a chromosomal translocation t(9;22)(q34;q11.2), which results in a fused oncogene called *BCR-ABL1* on the Philadelphia chromosome (1). Over the past 30 years, the global incident cases of CML have increased from 42.7 × 10^3^ in 1990 to 65.8 × 10^3^ in 2019 (2). The emergence of tyrosine kinase inhibitors (TKI) has significantly improved survival in CML; most patients treated with imatinib (a type of TKI) achieve normal life expectancy, with some patients achieving sustained treatment-free remission (TFR) (3). Globally, the age at CML diagnosis is decreasing, with the median age of approximately 56 to 57 years (4, 5); in some developing countries, the average age at diagnosis is <50 years (6). As the age of diagnosis decreases and survival improves, there is a need for effective CML management in pregnancy. However, the current state of research remains unclear.

Bibliometric analysis applies mathematical and statistical methods to quantitatively analyze all carriers of knowledge. It can be used with medical literature to evaluate any specific research domain. However, few bibliometric studies have been conducted in the field of CML research, and there are no bibliometric studies in the field of CML and pregnancy.

This study aimed to conduct a comprehensive bibliometric analysis of research literature published on CML and pregnancy over the past 20 years. Keywords, citation reports, number of annual publications, countries of study origin, international collaborations, authors, institutions, and journal titles were visually analyzed to elucidate the past and present trends in research, including key insights and areas of focus. The present findings may provide a comprehensive overview of the state of the field, informing future research in the management of CML in pregnancy.

## Materials and Methods

### Data Source and Search Strategy

A comprehensive search was performed using the Science Citation Index-Expanded of the Web of Science Core Collection. All searches were performed on the same day to avoid the significant bias caused by database updates. Two investigators (YW and LJ) independently performed the search and data extraction on October 13, 2021. The retrieval strategy was TS = [(“chronic myelogenous leukemia” OR “chronic myeloid leukemia” OR “chronic granulocytic leukemia” OR “chronic myelocytic leukemia” OR CML) AND (pregnancy OR pregnancies OR gestational OR reproduction OR gestational OR infertility)]. The period of interest was from 2001 to 2020. Only English language publications were included; the data category was limited to “article” and “review”.

### Software of Social Network Maps

VOS Viewer 1.6.15 (Leiden University, Leiden, Netherlands) (7, 8) is a software developed for building and visualizing bibliometric networks, here, it was used to visualize author collaborations and co–occurrence of keywords (9). “full counting” was the counting method. In the visual map, different nodes represented authors, countries, institutions, and keywords. The node size represented the corresponding number or frequency of reference. The links between nodes represented cooperation and co–occurrence relationships. The colors of the nodes and lines represented different clusters or corresponding years or average references.

We also used CiteSpace (5.8.R3), a visual knowledge graph bibliometric tool based on the Java language, to analyze the development dynamics and future trends in specific topics. This analysis focuses on finding critical points in the development of a field or domain, specifically, intellectual turning points and pivotal points. We used CiteSpace to visualize international collaborations among countries and institutions and visualize the co–citation of references, defined as a co–appearance of two articles in the bibliography of a third cited article. Findings were presented as clusters, according to the co–citation. In addition, we identified studies that experienced the greatest changes in the frequency of citations over a certain period. When an increase in the number of citations was detected for a particular study, the corresponding period was considered a period of popularity.

In addition, we used R–bibliometrix to perform descriptive analysis of the leading research countries and journals, including a historical direct citation network (10). We used pie charts to visualize the scientific output of countries.

Data were saved as plain text, and tab–delimited (Win, UTF–8) records with full record and cited references saved in WoSCC and imported into the relevant software, as required.

### Statistical Analysis

Python and Microsoft Office Excel 2007 were used for descriptive statistical analysis.

## Results

### Search Results

**Figure 1:**
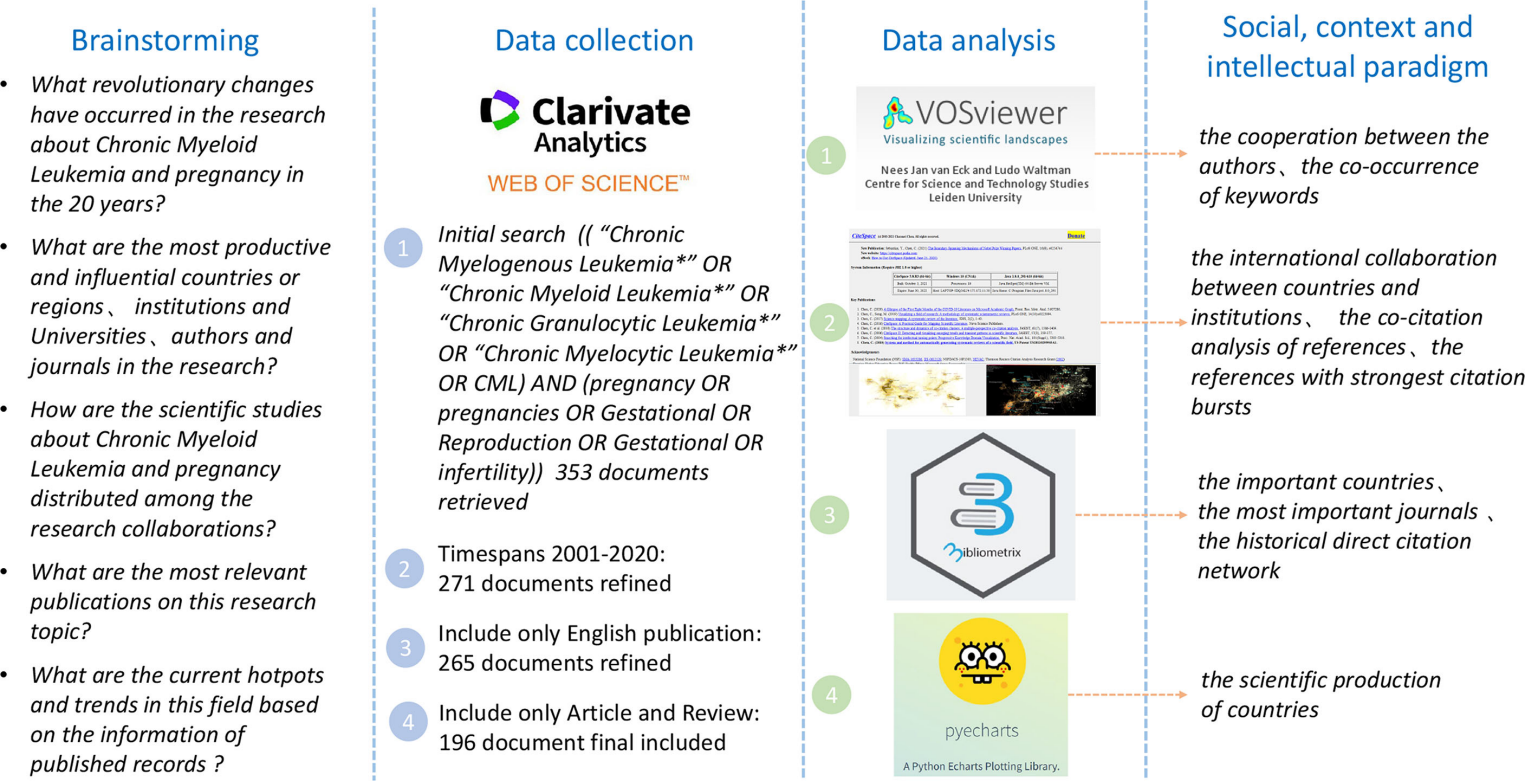
Conceptual design of the study.

The entire search retrieved a total of 353 publications. However, this study only included articles published from 2001 to 2020. Moreover, non–English language, non–article, and non–review publications were excluded. Finally, a total of 196 studies (154 articles and 42 reviews) were included in the bibliometric analysis. The study flow chart is presented in **Figure 1**.

### Trends and Annual Publications

**Figure 2:**
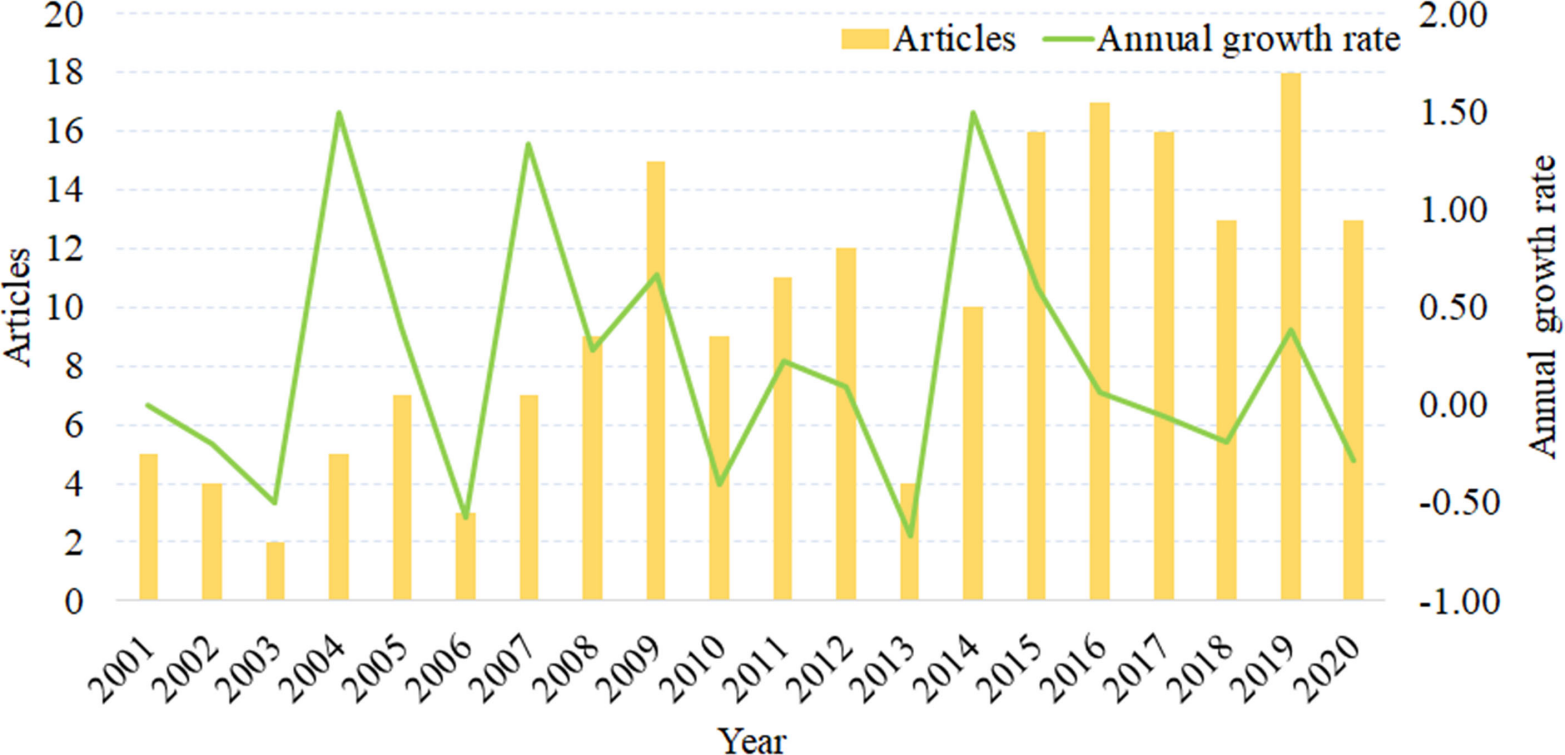
Literature growth curve (2001 to 2020).

There were wave–like fluctuations in the number of studies published and the annual growth rate of the body of literature (**Figure 2**). During the study period, the greatest and smallest numbers of studies were published in 2019 (18 articles) and 2003 (2 articles), respectively. There has been a significant increase in the number of publications in the last 6 years compared to the previous years.

### Analysis of Country and Region Output

**Figure 3:**
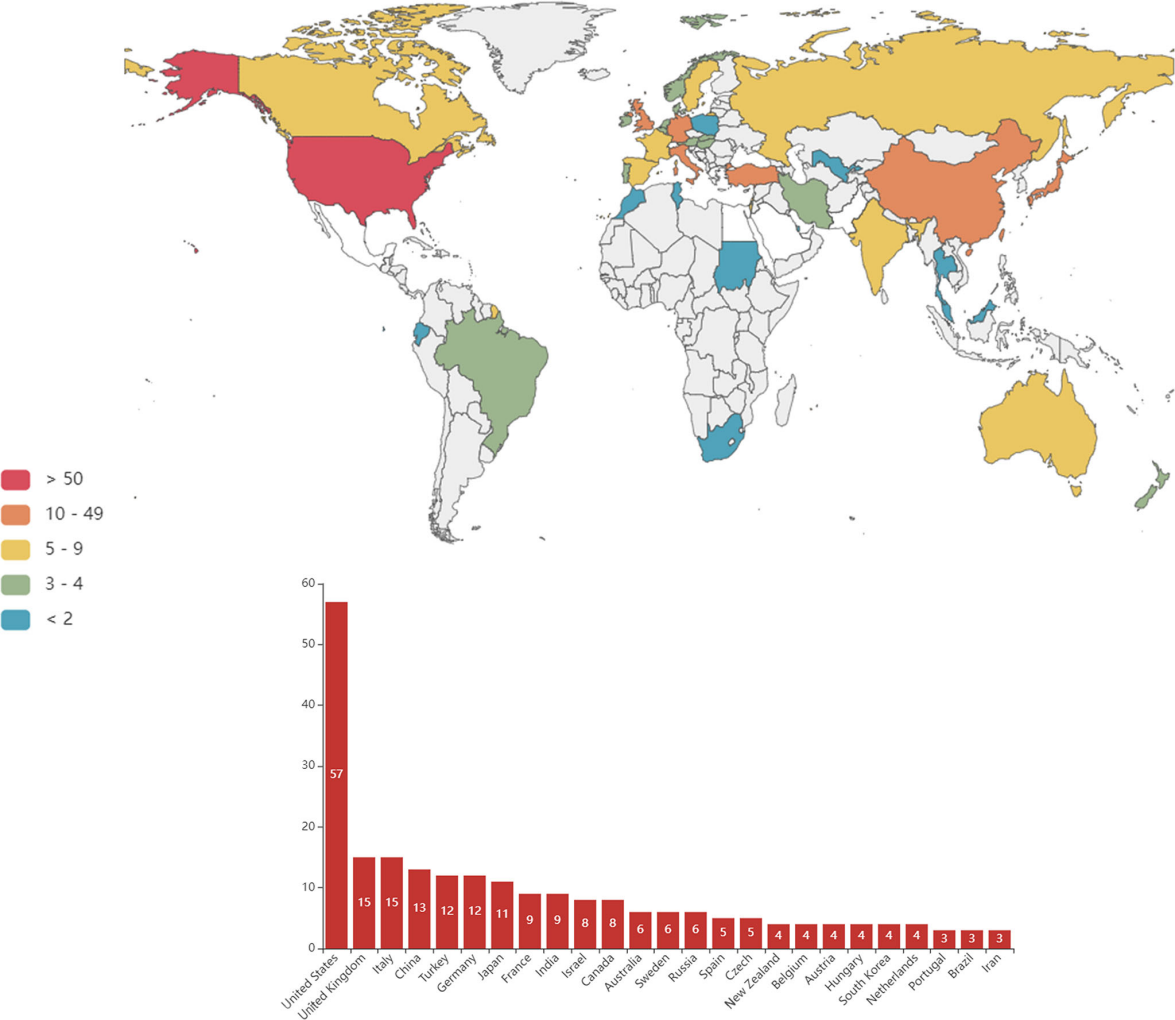
Countries/regions with research into chronic myeloid leukemia and pregnancy.

**Figure 4:**
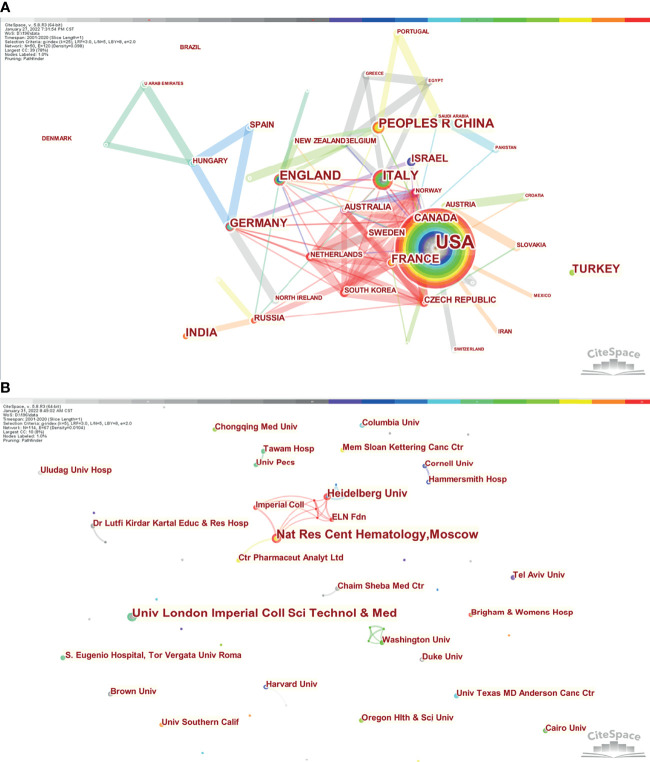
Co–authorship network of countries, regions, and institutions with chronic myeloid leukemia in pregnancy research. **(A)** Network visualization of countries/regions collaboration. **(B)** Network visualization of institutions/universities collaboration.

A total of 50 countries or regions contributed all publications on CML and pregnancy. Seven countries or regions contributed more than 10 publications, and 43 countries or regions contributed less than 10 documents (**Figure 3**). The United States (USA) (n=57, 29.1%) contributed the largest number of studies, accounting for nearly a quarter of the included studies. the United Kingdom (n=15, 7.7%), Italy (n=15, 7.7%), China (n=13, 6.6%), Turkey (n=12, 6.1%), Germany (n=12, 6.1%), and Japan (n=11, 5.6%) were the remaining top publishing countries (**Figure 3**). Global collaborations among countries and regions are presented in **Figure 4A**.

### Analysis of Institutional Output

This study included 421institutions, which made relevant contributions. Institutional collaborations among the producers of the top 114 publications are presented in **Figure 4B** and **Supplementary Table S1**. There were relatively few collaborations among institutions. Imperial College London and National Research Center for Hematology published the greatest number of articles (n=4), followed by Heidelberg University (n=3). Other institutions contributed 1–2 articles.

### Analysis of Authorship

**Figure 5:**
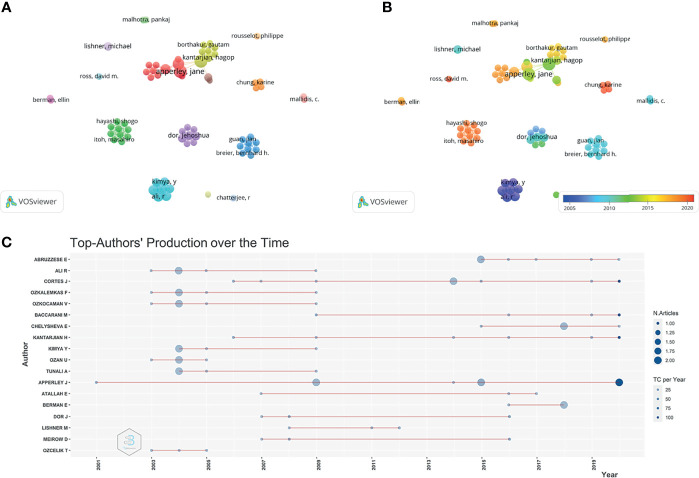
Co–authorship and co–citation analyses for chronic myeloid leukemia in pregnancy research. **(A)** Network visualization of authors co–authorship. **(B)** Overlay visualization of co–authorships. **(C)** Top authors’ publication record over time.

A total of 1,105 authors contributed relevant publications. A network map and overlay visualization of 70 co–authors who published more than 2 articles are presented in **Figures 5A** **, B**. These scholars were from different groups, the top 10 most productive and most–cited authors are presented in **Table 1**.

**Table 1:** Top 10 most relevant authors and most co–citation authors of Chronic Myeloid Leukemia and pregnancy research field.

Rank	Most Productive Authors (rank by Number)	Articles	Citations	Most Productive Authors (rank by Citation)	Citations	Articles
1	APPERLEY J	8	665	APPERLEY J	665	8
2	CORTES J	8	504	CORTES J	504	8
3	KANTARJIAN H	6	471	KANTARJIAN H	471	6
4	ABRUZZESE E	6	129	BACCARANI M	441	4
5	ALI R	5	181	DOR J	403	3
6	OZKALEMKAS F	5	181	MEIROW D	403	3
7	OZKOCAMAN V	5	181	SCHIFF E	403	3
8	BACCARANI M	4	441	AMARIGLIO N	339	2
9	KIMYA Y	4	151	RA'ANANI H	339	2
10	TUNALI A	4	151	BEN YEHUDA D	288	2

Apperley J (n=8) and Cortes J (n=8) published the highest number of manuscripts, followed by Abruzzese E (n=6), Kantarjian H (n=6). Citations are used to measure a researcher’s standing in the scientific community (11). Furthermore, Apperley J and Cortes J were the authors with the highest total number of citations (665 and 504, respectively, **Table 1**). Besides Apperley J started to study CML and pregnancy–related topics in 2001 and continued to publish in 2020 (**Figure 5C**). Abruzzese E had the highest number of publications in the past 5 years.

### Analysis of Journal Output

A total of 137 journals published relevant studies. *Leukemia Lymphoma* (n=7, United Kingdom) and *Leukemia Research* (n=7, United Kingdom) were the leading publishers of relevant articles. Meanwhile, the top three most–cited journals were *Blood* (750 citations, USA), *Journal of Clinical Oncology* (316 citations, USA), and *New England Journal of Medicine* (231 citations, USA) (**Table 2**).

**Table 2:** Distribution by top journals and its citations.

Rank	Top productive journals			Top–cited journals		
	Journals (IF&Q)	Articles	Country	Journals (IF&Q)	Total Citations	Country
1	LEUKEMIA LYMPHOMA (3.28, Q2)	7	United Kingdom	BLOOD (22.113, Q1)	750	USA
2	LEUKEMIA RESEARCH (3.152, Q2)	7	United Kingdom	J CLIN ONCOL (44.544, Q1)	316	USA
3	JOURNAL OF ASSISTED REPRODUCTION AND GENETICS (3.412, Q1)	5	USA	NEW ENGL J MED (91.245, Q1)	231	USA
4	AMERICAN JOURNAL OF HEMATOLOGY (10.047, Q1)	4	USA	LEUKEMIA (11.528, Q1)	218	United Kingdom
5	PLOS ONE (3.24, Q1)	4	USA	AM J HEMATOL (10.047, Q1)	211	USA
6	ACTA HAEMATOLOGICA (2.195, Q3)	3	Switzerland	BRIT J HAEMATOL (6.998, Q1)	163	United Kingdom
7	INTERNAL MEDICINE (1.271, Q3)	3	Japan	FERTIL AND STERIL (7.329, Q1)	148	Netherlands
8	INTERNATIONAL JOURNAL OF MOLECULAR SCIENCES (5.923, Q1)	3	Switzerland	LEUKEMIA LYMPHOMA (3.28, Q2)	135	United Kingdom
9	JOURNAL OF ONCOLOGY PHARMACY PRACTICE (1.809, Q3)	3	USA	LEUKEMIA RES (3.156, Q2)	750	United Kingdom
10	JOURNAL OF THE NATIONAL COMPREHENSIVE CANCER NETWORK (11.09, Q1)	3	USA	HUM REPROD (6.918, Q1)	316	United Kingdom

Bradford’s law, which is the law of diminishing returns and scattering, describes the relationship between a journal and the volume of articles and was first proposed in 1934 (12, 13). Here, this law was used to identify the most active journals. Our dataset had 21 zone core journals (**Supplementary Table S2**), which are the journals of most interest to researchers.

### Historical Evolution of CML and Pregnancy Research

**Figure 6:**
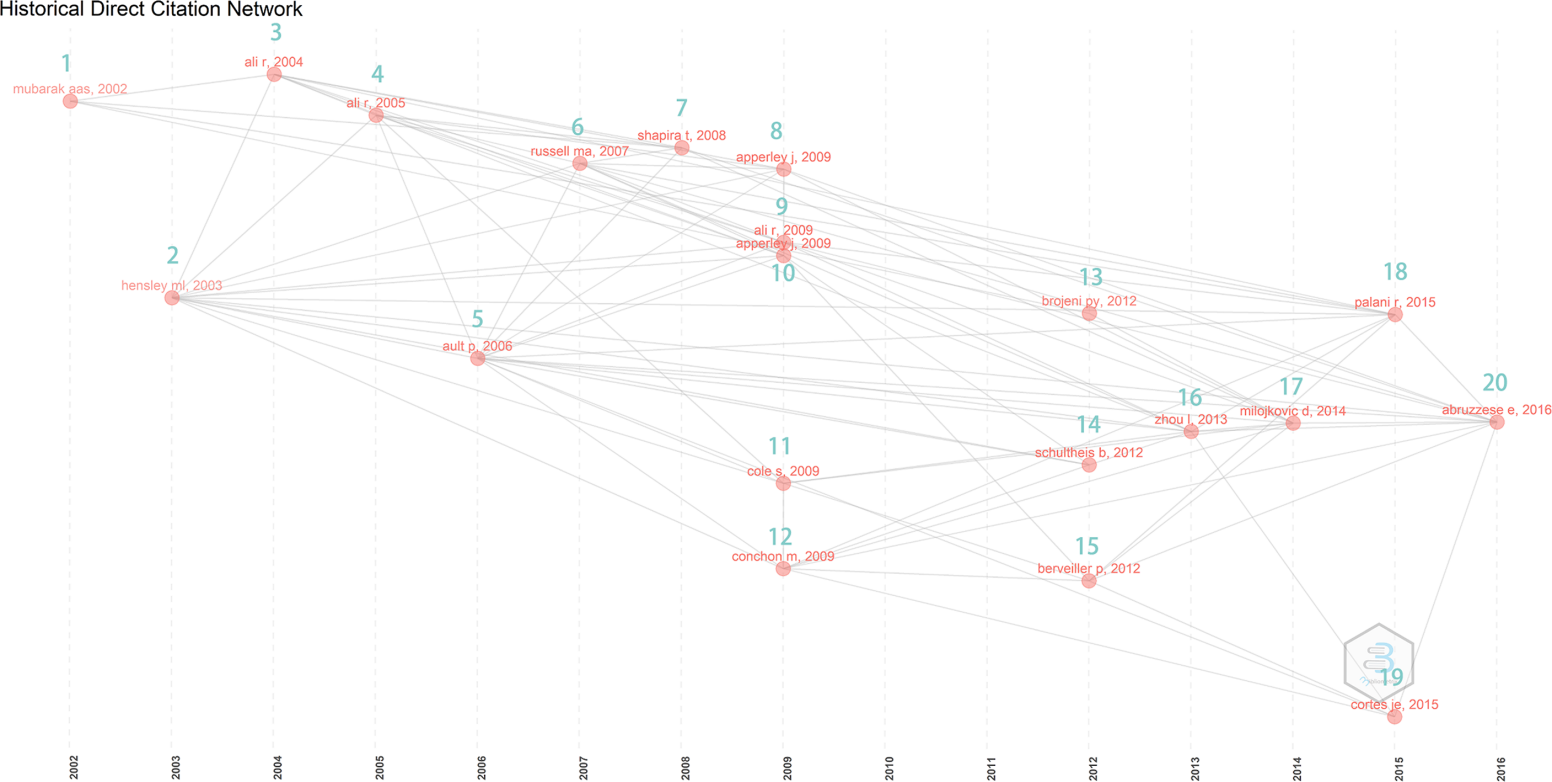
Historical direct citation network of chronic myeloid leukemia in pregnancy research.

To explore changes in the content of the relevant studies over time, we analyzed the historical direct citation network in CML and pregnancy research (**Figure 6** and **Table 3**). Treatment of CML during pregnancy was the focus of work initiated in 2002. With the introduction of TKI and improving survival rates, treatment effects on the fetus became increasingly relevant, resulting in an increase in the number of retrospective studies on this topic, starting with animal studies and case reports published in 2003. A study in 2009 showed no abnormality in children born to men taking imatinib, suggesting that treatment in men did not affect fertility. In 2009, there was an increase in the number of studies on CML treatment during pregnancy, showing that leukapheresis, hydroxyurea, and interferon could be used during pregnancy without resulting in fetal abnormalities, however, these findings were not validated. Some studies have found that delayed treatment, which has no impact on prognosis, may be an option for some patients. Subsequently, CML treatment during pregnancy became a research focus. Given treatment resistance and side effects associated with imatinib, the use of dasatinib is increasing, and its safety in pregnancy is being examined. A 2015 study has shown that dasatinib affects pregnancy outcomes, women treated with dasatinib are advised against continuing pregnancy. A 2016 study has recommended that female patients treated with TKI should be encouraged to use effective contraception due to the risk of fetal complications after drug exposure. Female patients should plan for pregnancy and discontinue TKI therapy. Individualized therapy needs to be considered when an unplanned pregnancy occurs.

**Table 3:** The historical direct citation network.

Rank	Title	Year	Journal	LCS	GCS
1	Normal outcome of pregnancy in chronic myeloid leukemia treated with interferon–alpha in 1st trimester: report of 3 cases and review of the literature	2002	AM J HEMATOL	18	37
2	Imatinib treatment: specific issues related to safety, fertility, and pregnancy	2003	SEMIN HEMATOL	34	126
3	Successful pregnancy and delivery in a patient with chronic myelogenous leukemia (cml), and management of cml with leukapheresis during pregnancy: a case report and review of the literature	2004	JPN J CLIN ONCOL	28	51
4	Pregnancy under treatment of imatinib and successful labor in a patient with chronic myelogenous leukemia (cml) – outcome of discontinuation of imatinib therapy after achieving a molecular remission	2005	LEUKEMIA RES	18	41
5	Pregnancy among patients with chronic myeloid leukemia treated with imatinib	2006	J CLIN ONCOL DOI	59	155
6	Imatinib mesylate and metabolite concentrations in maternal blood, umbilical cord blood, placenta and breast milk	2007	J PERINATOL	27	52
7	How I treat acute and chronic leukemia in pregnancy	2008	BLOOD REV	12	58
8	Issues of imatinib and pregnancy outcome	2009	J NATL COMPR CANC NE	10	34
9	Imatinib use during pregnancy and breast feeding: a case report and review of the literature	2009	ARCH GYNECOL OBSTET	14	42
10	CML in pregnancy and childhood	2009	BEST PRACT RES CL HA	14	50
11	Successful completion of pregnancy in a patient with chronic myeloid leukemia without active intervention: a case report and review of the literature	2009	CLIN LYMPHOMA MYELOM	20	27
12	Two successful pregnancies in a woman with chronic myeloid leukemia exposed to nilotinib during the first trimester of her second pregnancy: case study	2009	J HEMATOL ONCOL	23	45
13	A systematic review of the fetal safety of interferon alpha	2012	REPROD TOXICOL DOI	12	54
15	A dramatic fetal outcome following transplacental transfer of dasatinib	2012	ANTI–CANCER DRUG	15	33
14	Imatinib mesylate at therapeutic doses has no impact on folliculogenesis or spermatogenesis in a leukaemic mouse model	2012	LEUKEMIA RES	10	40
16	Pregnancies in patients with chronic myeloid leukemia treated with tyrosine kinase inhibitor	2013	LEUKEMIA RES	12	26
17	How I treat leukemia during pregnancy	2014	BLOOD	10	42
18	Managing pregnancy in chronic myeloid leukaemia	2015	ANN HEMATOL	18	48
19	The impact of dasatinib on pregnancy outcomes	2015	AM J HEMATOL	22	61
20	Management of pregnant chronic myeloidleukemia patients	2016	EXPERT REV HEMATOL	13	35

### Mutation Detection by Co–Citation Analysis

**Figure 7:**
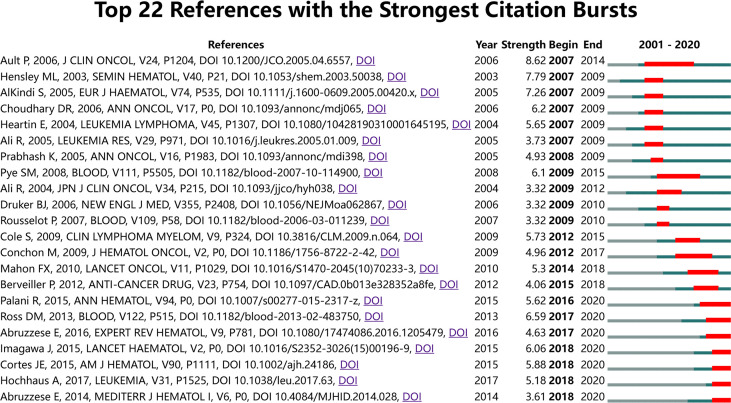
Mutation detection by co–citation analysis (outbreak durations≥2 years).

Co–citation analysis helps to understand past research hotspots and predict future research priorities for CML and pregnancy. References with outbreak durations greater than or equal to 2 years (2001–2020) are shown in **Figure 7** and **Supplementary Material S3**. The top 22 references with the highest number of citations are listed. The peak period of article citation tends to occur 3–4 years after initial publication. The first reference with a citation burst appeared in 2007, and several high–citation articles appeared in 2007, 2009, and 2018. In addition, several high–citation articles appeared in the last 3 years. The current research focus includes treatment impact on fertility, pregnancy, pregnancy outcomes, and disease outcomes in patients with CML treated with TKI.

### Conceptual Structural Map

**Figure 8:**
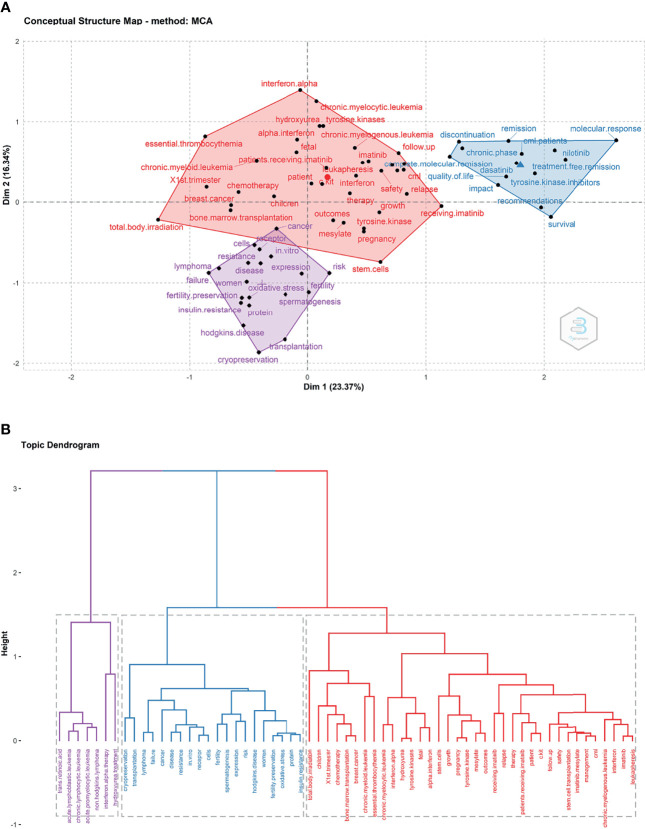
Structural map of chronic myeloid leukemia in pregnancy research fining sharing (2001 and 2020). **(A)** Conceptual structure map of publications. **(B)** Topic dendrogram.

A conceptual structure map of publications on CML and pregnancy revealed three topics of 36, 20, and 7 studies, focusing on patients who were or could become pregnant. These studies centered on CML treatment and its effects on the fetus (keywords: tyrosine kinase, mesylate, imatinib, chemotherapy, alpha interferon, hydroxyurea, total body irradiation, stem cells, bone marrow transplantation, stem cell transplantation, fetal, children, and outcomes), fertility preservation in CML patients (keywords: fertility preservation, cryopreservation, and spermatogenesis), CML disease status (keywords: treatment–free remission, complete molecular remission, molecular response, quality of life, and survival), Red topics are indicators of CML treatment countermeasures and their effects on the fetus, blue topics are indicators of CML disease status, purple topics are indicators related to the preservation of fertility in CML patients (**Figure 8** and **Supplementary Table S4**).

### Trends in CML and Pregnancy Research

**Figure 9:**
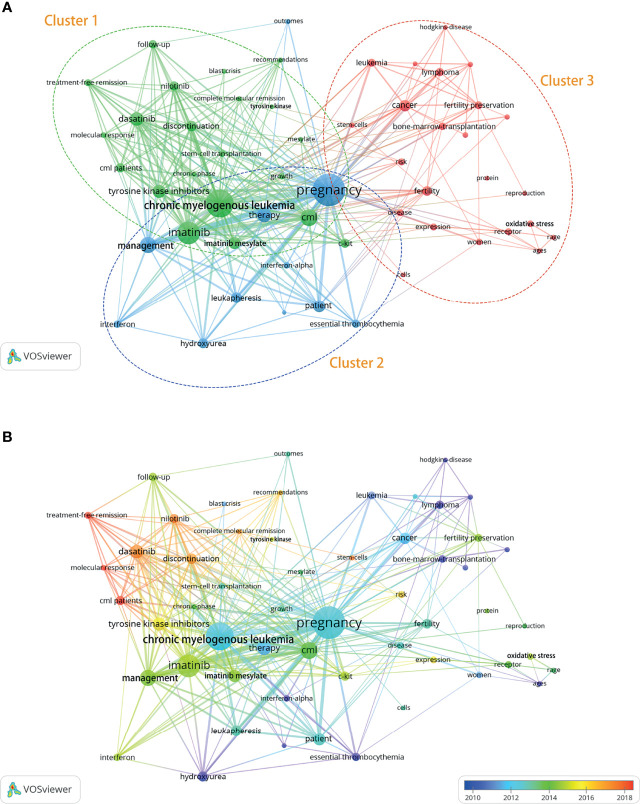
Distribution of keywords (frequency >5). **(A)** Network map of keyword frequency. **(B)** Trends in keyword frequency over time.

A total of 977 keywords were extracted from 196 published records. The keywords that occurred more than five times were organized into three clusters (**Figure 9A** and **Supplementary Table S5**). The three most used keywords were “chronic myelogenous leukemia”, “pregnancy”, and “imatinib”. Cluster 1, “chronic myelogenous leukemia” is the largest node and represents the current research focus. The main research focus includes imatinib mesylate, imatinib, dasatinib, nilotinib, tyrosine kinase inhibitors, cml, complete molecular remission, treatment–free remission, molecular response, discontinuation, chronic phase, blast crisis, growth, and follow–up. This cluster of topics is likely to become a research hotspot in the future. Cluster 2, “Unplanned pregnancy and CML management during pregnancy” includes topics such as pregnancy, management, therapy, leukapheresis, hydroxyurea, interferon, and interferon–alpha, all of which are gaining increasing attention. Cluster 3, “fertility preservation in CML patients” includes patients eligible for fertility preservation, associated techniques and influencing factors, such as women, ages, fertility, fertility preservation, cells, stem–cells, oxidative stress, and bone–marrow–transplantation. **Figure 9B** presents the change in keywords over time. The frequency of keywords such as imatinib, therapy, chronic–phase, follow–up and management gradually increased after 2014. Since 2018, the use of keywords such as treatment–free remission and complete molecular remission has increased, these areas are the current research hotspots. Future research may focus on the safe timing of pregnancy, pregnancy management, and outcomes in CML patients.

## Discussion

As the volume of research increases, bibliometric analysis can help identify data characteristics and patterns in any field of interest, capturing the key trends. In addition, bibliometric analysis can help visualize collaborations among countries, institutions, and authors, showing a chronological distribution of citations and milestone manuscripts. This study captured global collaboration and other research trends in the field of CML and pregnancy in the past 20 years.

A total of 196 records, published in 137 journals by 1,105 authors at 421 institutions in 50 countries and regions, were included in the analysis, revealing wave–like fluctuations. The number of publications in a research field indicates the scientific level of a country or institution (14). The USA was the leader in the number of publications, showing its influence in this field. In addition, the most influential institutions in the field of CML and pregnancy research were Imperial College London and National Research Center for Hematology. Finally, Apperley J, Cortes J, Kantarjian H, and Abruzzese E were the leading authors who might determine the focus and direction of research. However, the frequency of citations and the number of publications in no way detract from the value of the results of those authors who have few publications. The contribution is not always determined by quantitative indicators. Collaboration is paramount to research, few collaborations may account for the relatively few studies in CML and pregnancy research.

Few researchers are aware of all relevant journals in their field, and many struggle to select the most suitable outlet for their study. Journal indicators obtained from the bibliometric analysis can inform this decision (15). *Leukemia Lymphoma*, *Leukemia Research*, and the *Journal of Assisted Reproduction and Genetics* have published several articles on CML and pregnancy, making them zone core journals. Meanwhile, *Blood*, *Journal of Clinical Oncology*, and *New England Journal of Medicine* were the most frequently cited journals, scholars may consider these priority journals. Other journal output indicators may help inform submission decisions (**Table 2**).

Co–citation analysis helps examine the conceptual structure of research (16). The present conceptual structure maps, obtained by multiple correspondence analysis and K–means clustering, revealed three topics (**Figure 8**).

The topics included CML treatment during pregnancy and its effects on the fetus, fertility preservation in this patient group, and CML diagnosis, treatment, and disease status. The red topics captured CML treatment countermeasures and their effects on the fetus, the blue topics captured CML disease status, and the purple topics captured fertility preservation methods in this patient group.

Before 1950, arsenic and splenic irradiation were used for the palliative treatment of CML (17). Since the 1950s, chemotherapeutic agents such as hydroxyurea and busulfan have been used to control the disease without significantly changing the associated outcomes (18). Since the late 1970s, CML has been treated with allogeneic bone marrow or stem cell transplantation, which was associated with relatively low efficacy while being indicated only for young and otherwise healthy patients with a suitable match (19). Meanwhile, the 1980s saw the introduction of IFN–α to treat CML. However, IFN–α treatment had side effects such as fever and chills (20). In 2001, TKI was authorized by the Food and Drug Administration to treat CML, substantially improving survival rates (21). Due to the worldwide access to very efficacious treatments as well as the fact that young people are not treated routinely with transplant and do not die as before, the prevalence was increasing, and more pregnancies were reported over the past 20 years. Meanwhile, CML treatment is increasingly individualized. There are no guidelines on pregnancy in CML. Keyword co–occurrence analysis helps capture the present and future research trends. We identified three clusters of CML and pregnancy research:

### Cluster 1: Planned Pregnancy

For female patients, most studies concluded that there was a risk associated with increased miscarriage and malformations with TKI administration. Female patients during TKI treatment are strongly discouraged from attempting pregnancy and they should discontinue TKI treatment before conception. Since the first pilot study on TKI discontinuation published in 2007 by Rousselot (22), several prospective or retrospective discontinuation clinical trials have shown that some patients who achieved sustained deep molecular response (DMR) on long–term TKI therapy were able to achieve long–term treatment free remission (TFR), and thus TFR was a safe procedure to try in selected patients. When female patients achieve TFR, they can safely discontinue the TKI and try to get pregnant. There was some difference in the published TFR studies and related guidelines regarding the criteria for discontinuing TKI therapy. The National Comprehensive Cancer Network guidelines (2020) have determined the criteria for discontinuing TKI therapy (23): Age>18, chronic phase (CP) only, Duration of TKI therapy >5 years, Quantifiable typical transcript, A sustained DMR(MR4)>2 years, No prior history of progression or treatment resistance. The duration of TKI treatment, *BCR–ABL1* transcript and MR status have different criteria for recommending discontinuation as indicated in different guidelines (24). We need to take note that patients should be carefully selected, discontinuation should be done in a well–informed manner, and it was recommended that it must be done in highly specialized centers that are equipped to monitor and have the guidance from experienced physicians, with good regular follow–up and monitoring. MR levels are the primary method of assessing CML treatment response and obtaining molecular data, which can inform disease prognostication and treatment (25, 26). NCCN guidelines recommended monthly monitoring for the first year after discontinuation, then every six weeks for the second year, and every 12 weeks for the next year. Pregnancy can be planned when TFR is achieved in female patients discontinuing TKI therapy, but the optimal time for pregnancy is unclear (26). And numerous clinical trials are still needed to determine when the optimal time for pregnancy is achieved.

For female patients, planned pregnancies should be managed individually with tailored follow–up protocols when they stopped TKI therapy. NCCN guidelines recommended BCR–ABL levels are measured every 4 week 15 if *BCR–ABL* transcript levels rise above 1%. Some experts also recommended *BCR–ABL* levels need to be monitored every 4 weeks during pregnancy, and patients who have an elevated molecular response need frequent testing (27). If MMR is lost, management is determined by their clinical condition. It is currently thought that even if MMR is lost, patients who have reached TFR rarely experience rapid disease progression and therefore treatment may be delayed until the birth of the infant. If disease progression does accelerate, we need to treat accordingly. There are no studies that clearly describe the management of these patients, and the corresponding studies need to be further explored.

For female patients, most studies recommend that pregnancy is prohibited in the female patients of the accelerated phase or the blast crisis phase. If the female patients of the chronic phase fail to achieve sustained MMR or DMR but wishes to become pregnant, a change to TKIs may help achieve DMR, meanwhile, pregnancy should be postponed. These treatment modalities are suitable for chronic CML. Several studies have shown that switching from imatinib to 2G–TKI shortened the time between DMR and TFR, so some studies have suggested the use of 2GTKI as a first–line treatment option for female patients with CML–CP who desire pregnancy (28). This view needs further validation because of the increased cost of 2G–TKI, the severe side effects, and the lack of extensive clinical data.

For male patients, there was little effect on fertility and infant outcomes in male patients taking imatinib, bosutinib, dasatinib or nilotinib, as well as no significant increase in miscarriage rates in female partners (29–32), even though available clinical data suggested that TKI treatment may transiently affect androgens (33). However, experience is limited and the male patients taking TKI drugs may consider sperm freezing prior to starting TKI therapy.

### Cluster 2: Unplanned Pregnancy and CML Management During Pregnancy

Some patients do not meet the criteria for discontinuation at the time of pregnancy. A personalized approach is required in such cases, given the timing of diagnosis, treatment, and MR levels. Most studies currently consider that it is possible to continue the pregnancy as long as the CML status allows. The challenge in CML management during pregnancy is in balancing treatment–associated risks to the fetus with the potential benefits to the mother. With a fully informed choice to keep the fetus, the patient should immediately interrupt TKI therapy and closely monitor the disease status. For the maternal side, whether the interruption of treatment leads to disease progression [especially high residual leukemic load (≦̸MR2)], in addition CML leads to an increased risk of thrombosis and bleeding during pregnancy, which result in an elevated incidence of obstetric complications. For the fetus, it can cause placental insufficiency, intrauterine growth retardation, and serious risk of intrauterine death. When the risk of pregnancy increases, treatment during pregnancy is beneficial and necessary for both the patient and the fetus. The adverse effects of delayed treatment in pregnant patients have not been clearly reported in the literature, however, a few cases of rapid disease progression and eventual death have been reported (27).

Patients who become pregnant during CML treatment and at risk of hematologic or cytogenetic relapse require further treatment during pregnancy. Treatment modalities considered safe during pregnancy include leukapheresis and IFN. Nevertheless, for patients in the early pregnancy, it is preferable that no treatment is needed. The use of leukapheresis is limited because it is a temporary procedure that lowers the white blood cell count with no lasting effect. Patients with thrombocytosis may be considered for low–dose aspirin (not recommended in early pregnancy) or low–molecular heparin during pregnancy, but coagulation, liver function and other relevant indicators should be closely tested during the drug administration (34, 35). IFN is generally considered safe for the fetus and can be used before 15 weeks of gestation to achieve disease control without risking fetal organ development (36, 37). The data about peginterferon alfa–2a (PEG–IFNα) used in pregnancy were insufficient, thus we should be cautious in applying PEG–IFNα during pregnancy. The efficacy of hydroxycarbamide has been documented, however, the evidence is insufficient to recommend it for general use in pregnancy, although some case reports have shown no clear teratogenicity in the fetus (38, 39). The general recommendation regarding treatment in pregnancy is that TKI therapy should be avoided due to the increased risk of teratogenesis, especially during organogenesis (before 15 weeks of gestation). If TKI therapy is considered in mid– to late–pregnancy, it must be initiated with adequate information about the potential benefits of TKI therapy for the mother and the potential risks to the fetus. Some studies have shown that *BCR–ABL* levels should be measured in week 15. If *BCR–ABL* transcript levels rise above 1%–10%, TKI therapy may be considered, with a preference for imatinib (27). Dasatinib should not be used at any time during pregnancy (30). However, safety data from large studies in mid– to late–pregnancy are lacking, and the conclusion is not accepted by most experts.

### Cluster 3: Fertility Preservation in CML Patients

Fertility preservation methods include semen cryopreservation, oocyte extraction and storage, and embryo cryopreservation (40, 41). Although current research has shown that spontaneous pregnancy after TKI treatment is possible, fertility preservation could be discussed with both male and female patients of childbearing age scheduled to undergo TKI therapy, as some studies shown that it may affects oocyte and sperm maturation and the overall gonadal function and pregnancy failure increases with age. Imatinib resistance correlates with oxidative stress in CML patients (42). Hematopoietic stem cell transplantation can be used to treat CML in patients ineligible for TKI therapy, although this is not a first–line treatment option. However, hematopoietic stem cell transplantation is highly gonadotoxic as high–dose chemotherapy and total body irradiation were required for hematopoietic stem cell transplantation (43). Ovarian stimulation and embryo freezing may be performed before transplantation to preserve fertility, and IFN replacement therapy is recommended to help promote ovulation and preserve fertility (44).

To the best of our knowledge, this is the first bibliometric analysis of CML and pregnancy research. However, this study had some limitations. First, most of our analysis was based on the Web of Science Core Collection database, which is constantly updated, making the present findings temporary in nature. Second, this study included only English–language original articles and reviews. Third, there are not enough articles on CML and pregnancy, thus there is some bias in the results. Fourth, it may not have been enough time for some articles to be read and cited by interested authors when the comprehensive search was performed. However, this study aimed to conduct a high–quality bibliometric analysis of CML in pregnancy research, consequently, these limitations are unlikely to affect the present findings, which capture research trends in the field of interest.

In summary, the present study reviewed trends in research into CML in pregnancy from 2001 to 2020, revealing the leading research countries, institutions, and authors, as well as the present and future key topics. This field of research has presented wave–like fluctuations. The USA emerged as a research leader. Imperial College London and National Research Center for Hematology were the most influential institutions in this field. Apperley J, Cortes J, Abruzzese E and Kantarjian Hare the leading authors, likely determining research focus. The keywords were organized into four clusters, including planned pregnancy, unplanned pregnancy and CML management during pregnancy, and fertility preservation in CML patients. Relatively few collaborations may account for the low number of articles published in this field. These findings may help inform authors’ submission strategy and future research trends.

## Data Availability Statement

‘The original contributions presented in the study are included in the article/**Supplementary Material**. Further inquiries can be directed to the corresponding author.

## Author Contributions

YZ conceived the study and critically revised the content of this manuscript. YW and LJ made significant contributions to the study methods, results, and interpretation. BL was involved in the design and writing of the manuscript. All authors contributed to the article and approved the submitted version.

## Funding

This work was supported by grants from the Science and Technology Projects of Liaoning Province (2021JH2/10300093).

## Conflict of Interest

The authors declare that the research was conducted in the absence of any commercial or financial relationships that could be construed as a potential conflict of interest.

## Publisher’s Note

All claims expressed in this article are solely those of the authors and do not necessarily represent those of their affiliated organizations, or those of the publisher, the editors and the reviewers. Any product that may be evaluated in this article, or claim that may be made by its manufacturer, is not guaranteed or endorsed by the publisher.
