# Supplementary material for: Management of Chronic Myeloid Leukemia and Pregnancy: A Bibliometric Analysis (2000-2020)
Source: Front Oncol. 2022 Mar 7;12:826703. doi: 10.3389/fonc.2022.826703 (PMC8934933; doi:10.3389/fonc.2022.826703)
Supplement: Supplementary file 3 [file DataSheet_3.pdf]

Top 22 References with the Strongest Citation Bursts

References	Year	Strength	Begin	End	2001 - 2020
Ault P, 2006, J CLIN ONCOL, V24, P1204, DOI 10.1200/JCO.2005.04.6557, DOI	2006	8.62	**2007**	2014	
Hensley ML, 2003, SEMIN HEMATOL, V40, P21, DOI 10.1053/shem.2003.50038, DOI	2003	7.79	**2007**	2009	
AlKindi S, 2005, EUR J HAEMATOL, V74, P535, DOI 10.1111/j.1600-0609.2005.00420.x, DOI	2005	7.26	**2007**	2009	
Choudhary DR, 2006, ANN ONCOL, V17, P0, DOI 10.1093/annonc/mdj065, DOI	2006	6.2	**2007**	2009	
Heartin E, 2004, LEUKEMIA LYMPHOMA, V45, P1307, DOI 10.1080/10428190310001645195, DOI	2004	5.65	**2007**	2009	
Ali R, 2005, LEUKEMIA RES, V29, P971, DOI 10.1016/j.leukres.2005.01.009, DOI	2005	3.73	**2007**	2009	
Prabhash K, 2005, ANN ONCOL, V16, P1983, DOI 10.1093/annonc/mdi398, DOI	2005	4.93	**2008**	2009	
Pye SM, 2008, BLOOD, V111, P5505, DOI 10.1182/blood-2007-10-114900, DOI	2008	6.1	**2009**	2015	
Ali R, 2004, JPN J CLIN ONCOL, V34, P215, DOI 10.1093/jjco/hyh038, DOI	2004	3.32	**2009**	2012	
Druker BJ, 2006, NEW ENGL J MED, V355, P2408, DOI 10.1056/NEJMoa062867, DOI	2006	3.32	**2009**	2010	
Rousselot P, 2007, BLOOD, V109, P58, DOI 10.1182/blood-2006-03-011239, DOI	2007	3.32	**2009**	2010	
Cole S, 2009, CLIN LYMPHOMA MYELOM, V9, P324, DOI 10.3816/CLM.2009.n.064, DOI	2009	5.73	**2012**	2015	
Conchon M, 2009, J HEMATOL ONCOL, V2, P0, DOI 10.1186/1756-8722-2-42, DOI	2009	4.96	**2012**	2017	
Mahon FX, 2010, LANCET ONCOL, V11, P1029, DOI 10.1016/S1470-2045(10)70233-3, DOI	2010	5.3	**2014**	2018	
Berveiller P, 2012, ANTI-CANCER DRUG, V23, P754, DOI 10.1097/CAD.0b013e328352a8fe, DOI	2012	4.06	**2015**	2018	
Palani R, 2015, ANN HEMATOL, V94, P0, DOI 10.1007/s00277-015-2317-z, DOI	2015	5.62	**2016**	2020	
Ross DM, 2013, BLOOD, V122, P515, DOI 10.1182/blood-2013-02-483750, DOI	2013	6.59	**2017**	2020	
Abruzzese E, 2016, EXPERT REV HEMATOL, V9, P781, DOI 10.1080/17474086.2016.1205479, DOI	2016	4.63	**2017**	2020	
Imagawa J, 2015, LANCET HAEMATOL, V2, P0, DOI 10.1016/S2352-3026(15)00196-9, DOI	2015	6.06	**2018**	2020	
Cortes JE, 2015, AM J HEMATOL, V90, P1111, DOI 10.1002/ajh.24186, DOI	2015	5.88	**2018**	2020	
Hochhaus A, 2017, LEUKEMIA, V31, P1525, DOI 10.1038/leu.2017.63, DOI	2017	5.18	**2018**	2020	
Abruzzese E, 2014, MEDITERR J HEMATOL I, V6, P0, DOI 10.4084/MJHID.2014.028, DOI	2014	3.61	**2018**	2020	
